# Full-Mouth Rehabilitation of a 15-Year-Old Girl Affected by a Rare Hypoparathyroidism (Glial Cell Missing Homolog 2 Mutation): A 3-Year Follow-Up

**DOI:** 10.3390/dj12050130

**Published:** 2024-05-07

**Authors:** Yohann Flottes, Eléonore Valleron, Bruno Gogly, Claudine Wulfman, Elisabeth Dursun

**Affiliations:** 1 UFR Dentistry, Faculté de Santé, Université Paris Cité, 75006 Paris, France; yohann.flottes@u-paris.fr (Y.F.); eleonore.valleron@u-paris.fr (E.V.); bruno.gogly@u-paris.fr (B.G.); claudine.wulfman@u-paris.fr (C.W.); 2 Department of Dentistry, AP-HP, Henri Mondor Hospital, 94000 Créteil, France; 3 URB2i, Université Paris Cité, 92120 Montrouge, France

**Keywords:** hypoparathyroidism, fixed dental prosthesis, dental bonding, dental enamel hypoplasia

## Abstract

Objective: Familial isolated hypoparathyroidism is a rare genetic disorder due to no or low production of the parathyroid hormone, disturbing calcium and phosphate regulation. The resulting hypocalcemia may lead to dental abnormalities, such as enamel hypoplasia. The aim of this paper was to describe the full-mouth rehabilitation of a 15-year-old girl with chronic hypocalcemia due to a rare congenital hypoparathyroidism. Clinical considerations: In this patient, in the young adult dentition, conservative care was preferred. Onlays or stainless-steel crowns were performed on the posterior teeth, and direct or indirect (overlays and veneerlays) were performed on the maxillary premolars, canines, and incisors, using a digital wax-up. The mandibular incisors were bleached. The treatment clearly improved the patient’s oral quality of life, with fewer sensitivities, better chewing, and aesthetic satisfaction. The difficulties were the regular monitoring and the limited compliance of the patient. Conclusion: Despite no clinical feedback in the literature, generalized hypomineralized/hypoplastic teeth due to hypoparathyroidism in a young patient can be treated as amelogenesis imperfecta (generalized enamel defects) with a conservative approach for medium-term satisfactory results. Highlights: This study provides new insights into the management of enamel hypoplasia caused by familial isolated hypoparathyroidism, helping to improve patient outcomes in similar cases.

## 1. Introduction

Hypoparathyroidism is a metabolic disorder characterized by a deficiency or absence of parathyroid hormone secretion [1]. This disorder is most often acquired; but, more rarely, it can also be linked to a congenital or functional cause (Table 1) [2]. Parathyroid hormone (PTH), secreted by the parathyroid glands, plays a central role in the regulation of calcium and phosphorus metabolism [3].

Thus, calcium and phosphate levels are affected, and this may lead to hypocalcemia and hyperphosphatemia [4]. Hypocalcemia is an abnormality of ionized blood calcium, due to increased calcium loss or decreased calcium entry into circulation [5,6,7]. In its acute form, it can cause neuromuscular manifestations (paresthesia, muscle spasms, and epileptic seizures) or cardiac manifestations (ST-segment and QT prolongation without T-wave modification, atrioventricular blocks, and ventricular fibrillation) [8]. Hyperphosphatemia has no clinical symptoms of its own but contributes to hypocalcemia [9].

Many different but sporadically occurring dental features have been found in patients with hypoparathyroidism. A recent systematic review reported enamel hypoplasia (42%), enamel opacities (19%), hypodontia (12%), and various types of eruption disturbances (>6%) [10]. For the question of whether the management of generalized structural enamel defects, such as amelogenesis imperfecta, has already been reported, to our knowledge, no studies have reported oral care in patients. Moreover, the use of CAD-CAM makes it possible to perform long-lasting esthetic restorations in a short space of time.

The aim of this paper was to present a full-mouth rehabilitation with a strict conservative approach in a 15-year-old girl affected by a familial isolated hypoparathyroidism (glial cells missing transcription factor 2 mutation, GCM2).

## 2. Patient Presentation

### 2.1. Patient’s Information

**Figure 1:**
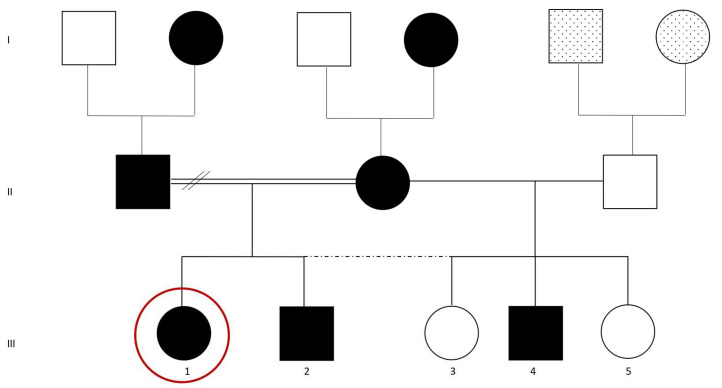
Patient’s family tree. The red circle represents the patient. Black color means “carrier of the mutation”. White color means “non carrier of the mutation. The point symbol means “no data”. Circles represent women/girls. Squares represent men/boys. The double line represents a link between consanguineous relatives. Dotted lines correspond to half-siblings. The two slashes correspond to partners now separated. Arabic numbers represent sibling order and Latin numbers represent the three generations.

A 15-year-old girl attended the Department of Dentistry (Henri Mondor Hospital, APHP, Creteil, France) complaining of chronic generalized dental and gingival pain when eating or drinking something cold, as well as brushing difficulties. The patient reported experiencing generalized hypersensitivity mainly while chewing and under pressure. Continuous pain was described without any specific pattern. Although the patient was shy and not very talkative, she expressed her concern about the unaesthetic appearance of her teeth. The patient’s history revealed that she was affected by a familial isolated hypoparathyroidism, caused by the glial cells missing transcription factor 2 (GCM2) gene mutations. Her mother and father were first cousins. Her mother and grandmother on the maternal side, as well as her father and her grandmother on the paternal side, carried the mutation. In addition, her brother and one of her half-brothers were also carriers (Figure 1).

Her hypoparathyroidism was first characterized by severe repeated hypocalcemia episodes in the neonatal period. Her medical chart reported difficulties in obtaining a correct calcium balance during childhood and adolescence, due to poor compliance with treatment. In addition, she also presented nephrocalcinosis without chronic renal failure. In April 2021, she was hospitalized for a new severe episode of hypocalcemia. Post-traumatic chronic pain syndrome was identified (with chronic headaches and abdominal pain), associated with an early depressive syndrome, chronic pelvic pain, and dysmenorrhea (without an identified gynecological cause); anorexia without weight loss with progressive food recovery; and microcytic anemia with inflammatory and deficiency components. She was treated with vitamin D, calcium, iron, and melatonin supplementation, teriparatide (an analogue of parathormone), amitriptyline, and alimemazine. The patient reported living with her mother due to a family conflict. Her schooling was interrupted by frequent hospitalizations. Her medical reports often reported noncompliance with therapy. She reported snacking frequently and not consuming enough fruits and vegetables. Regarding her family’s dental care, the patient’s grandmother has received treatment for a new complete denture. One of the patient’s brothers has been monitored for trauma to his 21, without other context of enamel hypoplasia. Unfortunately, all the patient’s relatives could not be examined.

### 2.2. Clinical Examination

**Figure 2:**
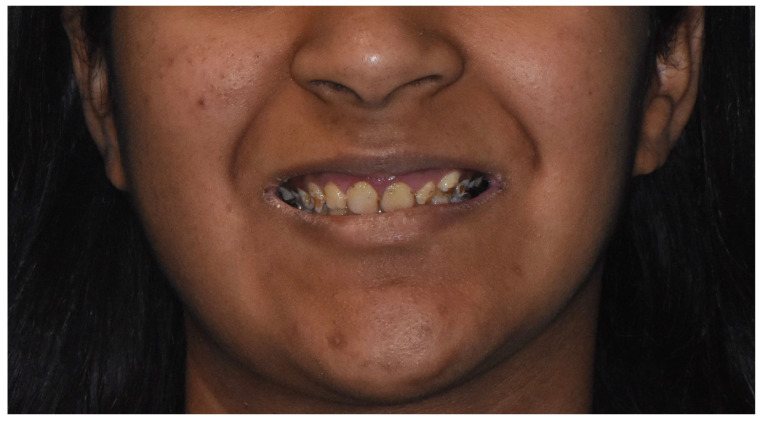
Initial extraoral view.

Extraoral examination revealed a slight decrease in the lower third of the face, associated with bilateral angular cheilitis and a forward chin (Figure 2).

**Figure 3:**
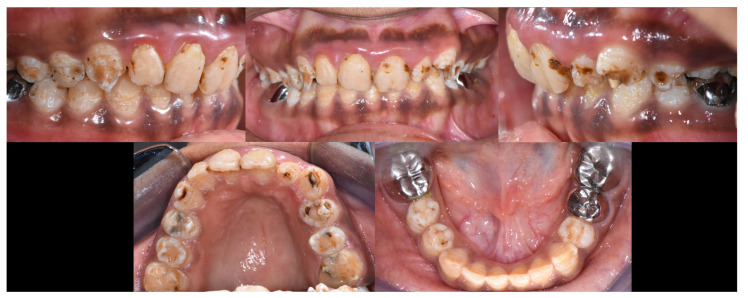
Initial intraoral views.

Intraoral examination (Figure 3) showed early permanent dentition with generalized loss of the tooth structure and previous treatments. In fact, the enamel was chalk-white to yellow-brown, with a striated and relatively friable structure, and decayed in some areas (on 13, 14, 15, 23, 24, 25). In addition, previous treatments were not satisfactory, such as stainless-steel crowns (SSC) (not respecting the occlusal curve on 35), inadequate resin composite restorations (16, 17, 26, 27, 47) on the posterior teeth, and resin composite restorations with secondary carious lesions on the anterior teeth (11, 12, 21, 22). Regarding the gingival status, the patient presented generalized gingival inflammation, plaque deposits with a Silness–Löe plaque index [11] close to 100%, calculus deposits, and gingival bleeding (during brushing and probing). Intra-arch examination showed U-shaped arches, the palato-version of 11 and 21, the labio-version of 23, and disruption of the Spee’s curve due to incorrect restoration on 35. Inter-arch examination showed a midline deviation but no posterior crossbite, a slight overbite, and loss of the posterior vertical dimension class II subdivision left malocclusion (Angle classification, [12]). However, the occlusion was stable.

### 2.3. Radiographic Examinations

**Figure 4:**
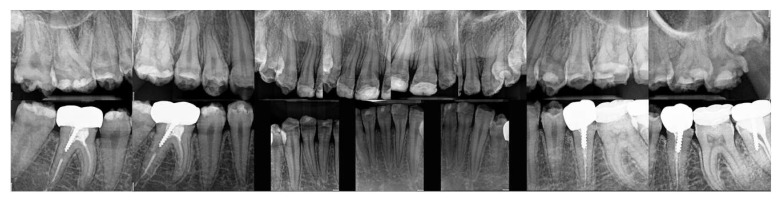
Initial radiographs.

Radiographs (Figure 4) showed a thin enamel, confirmed the inadequate restorations, and revealed secondary caries under the SSCs (35, 36, 37). Moreover, pulp stones were noted on 36, as well as defective endodontic treatments in density or length (35, 37, 46) without endodontic periodontal lesions and inadequate root anchorages (35, 37, 46).

### 2.4. Diagnostic and Therapeutic Proposal

The patient presented generalized gingivitis and enamel hypoplasia with a high caries risk (active lesions, enamel defects, and defective restorations). She had a strong aesthetic demand but a limited compliance (particularly with oral hygiene), directing us to priority care. The previous dental treatment was performed under general anaesthesia. Orthodontic treatment was offered to the patient as the first intention. This was refused by the patient because the large number of hospitalizations and intensive care stays was not necessarily compatible with therapy as serious as orthodontic treatment. In addition, the patient wanted “quick” and effective treatment. The following procedures were chosen:

- To stabilize the caries risk, we decided to improve the dental hygiene, and proceed to periodontal therapy;
- For the posterior maxillary and mandibular teeth, we decided not to intervene in the stable and acceptable restorations and to restore the others with direct/indirect resin composite, glass ionomer cement, or SSCs. The possibility of achieving a dry field and the thickness of the residual tissues were the main arguments for the choice of restoration type;
- For the anterior maxillary teeth, we decided to restore them with indirect resin composite overlays or veneers, according to the residual tissues;
- For the anterior mandibular teeth, we decided to treat them with teeth whitening;
- Lastly, to increase the lower third of the face, an increase of 2 mm in the vertical dimension was chosen.

The alternative treatment approach would have been to use SSCs instead of onlays on the maxillary premolars. This approach was not chosen for aesthetic reasons, as requested by the patient. The choice of an onlay on 16 was made for tissue preservation reasons.

### 2.5. Treatment Procedure

**Figure 5:**
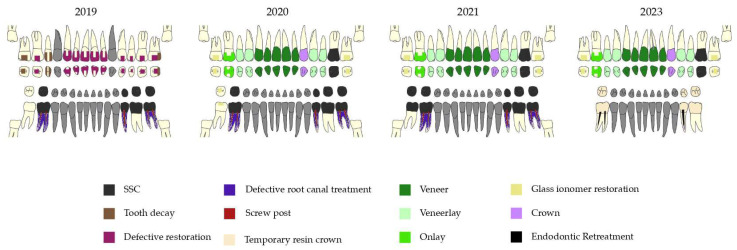
Treatment workflow over the years.

Treatment consisted of a preliminary session to study the case, followed by eight care sessions. The oral quality of life was measured with the General Oral Health Assessment Index (GOHAI [13]) before and at the end of the treatment, as well as at each follow-up session. The stages of treatment over the years are summarized in Figure 5.

### 2.6. Initial Therapy and Impressions for Digital Study Models (First Session)

#### 2.6.1. Oral Hygiene and Nutrition Education

The first step was oral hygiene education. The brushing defect was related to dental pain. A soft toothbrush, a bi-fluoride toothpaste, a bi-fluoride mouthwash, a casein Phosphopeptide—Amorphous Calcium Phosphate paste (ToothMousse^®^ by GC Corporation, Tokyo, Japan)—and dental floss were prescribed. The modified Stillman brushing technique was explained [14]. Re-evaluations at each session help to improve the plaque index. Moreover, a food diary was kept, which was then analyzed and associated with nutritional advice.

#### 2.6.2. Quality of Life Assessment

We chose the GOHAI as a self-assessment tool to evaluate the impact of dental health on daily life. This questionnaire is available in French, and it is composed of 12 items (rated from one to five). A score between 57 and 60 indicates good satisfaction, a score between 51 and 56 indicates average satisfaction, and a score less than 50 shows low satisfaction (10). At the beginning of the treatment, a score of 28 (low satisfaction) was recorded (Table 2).

#### 2.6.3. Intraoral Scanning for Digital Study Models

**Figure 6:**
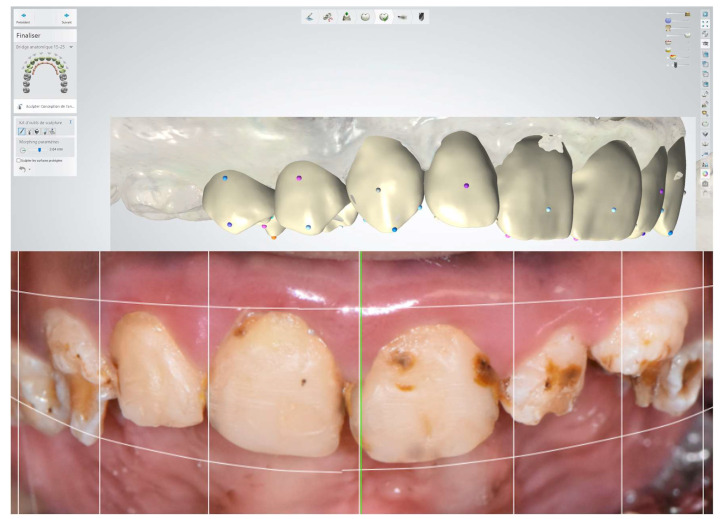
Virtual wax-up.

Intraoral scanning was performed (Trios 3 camera by 3Shape, Copenhagen, Denmark) to obtain digital study models. A virtual wax-up was created using a 3Shape^®^ computer-aided design and manufacturing system. The choice of the proportions of the future teeth was made by evaluating the shape of the face and the aesthetic possibilities (Digital Smile Design study with facial, extra, and intraoral photographs) (Figure 6). The digital models were printed to make the silicone key for mock-up (for the sixth session).

### 2.7. Management of the Posterior Mandibular Teeth (Second Session)

For the left mandibular first molar (36), the SSC was replaced and sealed with glass ionomer cement (Fuji Plus by GC Corporation, Tokyo, Japan). For the left mandibular second premolar (35), an occlusal adjustment on the SSC was indicated to obtain a right curve. To minimize the number of dental interventions, the SSCs on 37 and 46 and the restoration on 47, which were considered clinically acceptable, were not replaced.

### 2.8. Management of the Maxillary Molars (Third and Fourth Sessions)

#### 2.8.1. Third Session

**Figure 7:**
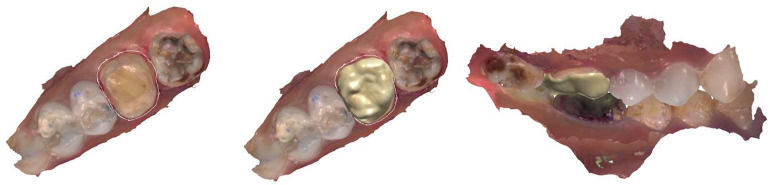
Onlay virtual design (for 16).

The two first permanent molars (16 and 26) were restored in the same session to increase the vertical dimension. For the right maxillary first molar (16), moisture control allowed the indication of a chairside CAD/CAM resin composite onlay in a polymer-infiltrated ceramic network (Vita Enamic^®^ Cerec 2M2-HT by VITA Zahnfabrik, BadSackingen, Germany) (Figure 7).

The previous restorations were removed, and the tooth was minimally prepared. The dental surface was sandblasted with 27 µm powder (RONDOflex^TM^ by KaVo, Bieberach an der Riss, Germany); then, 5% sodium hypochlorite was applied for one minute, after which it was rinsed with water, and a universal adhesive (VivaPen by Ivoclar, Schaan, Liechtenstein) was brushed on. The intaglio of the prosthetic restoration was etched with 9% hydrofluoric acid (Porcelain Etch by Ultradent Products, South Jordan, UT, USA) for one minute, thoroughly rinsed with water, and dried; then, silane was applied (Monobond Plus by Ivoclar, Schaan, Liechtenstein). Bonding was performed with a resin composite (Variolink^®^ Esthetic DC neutral by Ivoclar, Schaan, Liechtenstein). The light curing was carried out (Bluephase Style by Ivoclar, Schaan, Liechtenstein, 240 V, 1.100 W/cm^2^) for 40 s on each side. For the left maxillary first molar (26), the absence of moisture control and the thinness of the residual tissues led us to perform an SSC (3M^TM^, St Paul, MN, USA). The carious tissue was removed, the tooth was slightly prepared, and then the SSC was sealed with glass ionomer cement (Fuji Plus by GC Corporation, Tokyo, Japan).

#### 2.8.2. Fourth Session

For the second maxillary molars (17, 27), the low height of the clinical crown, the small loss of tissues, and the difficulty controlling the moisture led us to glass ionomer (EQUIA Forte^TM^ HT Fil by GC Corporation, Tokyo, Japan) restorations after caries removal.

### 2.9. Anterior Mandibular Teeth Whitening (Fifth Session)

**Figure 8:**
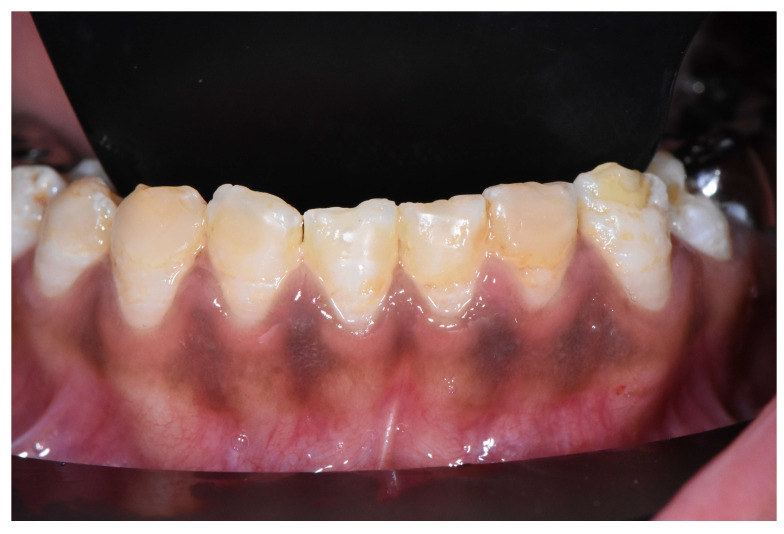
Mandibular view after whitening.

A conventional impression of the mandibular teeth was performed to obtain a soft transparent resin splint. Pola Night 10% carbamide peroxide (by Southern Dental Industries, Bayswater, Australia) was used for thirty minutes for the first day and then for one hour for twenty days. The treatment was well tolerated without sensitivity (Figure 8).

### 2.10. Rehabilitation of the Maxillary Second Right Premolars to Maxillary Second Left Premolars

#### 2.10.1. Sixth Session (Mock-Up Session)

A silicone key was made with ultra-light poly-addition silicone (Aquasil^®^ Ultra XLV by Dentsply-Sirona, Charlotte, NC, USA) and poly-addition silicone (Aquasil^®^ Hard Putty/Fast Set by Dentsply-Sirona, Charlotte, NC, USA) on the resin-printed model. This key was used to perform the mock-up in the chemo-polymerizable resin composite (Structur 2 SC A1 by VOCO, Cuxhaven, Germany).

#### 2.10.2. Seventh Session (Tooth Preparation)

**Figure 9:**
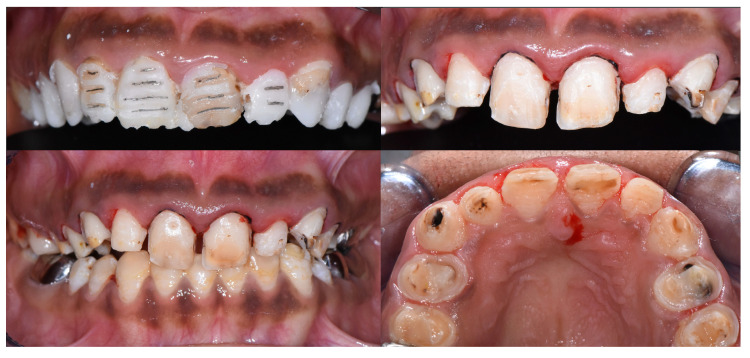
Intraoral views of veneer preparation.

Marks were drawn on the mock-up to indicate the required thickness for the veneer preparations. The teeth were prepared for us to perform the following (Figure 9):

- For all the premolars (15, 14, 24, 25), we performed overlays with a buccal juxtagingival margin.
- For the right maxillary canine (13), we performed a crown with total peripheral preparation in the juxtagingival margin.
- For the left maxillary canine (23), we performed a vestibular veneer with palatal return.
- For all the incisors (12, 11, 21, 22), we performed vestibular veneers with palatal return.

An intraoral scan of the preparations was performed. Then, a new temporary restoration was made after an immediate dental sealing (IDS) with a universal adhesive (ScotchBond^TM^ by 3M^TM^, St Paul, MN, USA).

#### 2.10.3. Eighth Session (Bonding)

All the onlays (15, 14, 13, 24, 25) and veneers (12, 11, 21, 22) were made of a polymer-infiltrated ceramic network (Vita Enamic^®^ Cerec 1M2-HT by VITA Zahnfabrik, BadSackingen, Germany) and bonded according to the same procedure described for the onlay on 16. The palatal surfaces of the incisors were also covered with resin composite (Herculite^TM^ XRV A2 and Optibond^TM^ by Kerr, Orange, CA, USA).

### 2.11. Follow-Ups

#### 2.11.1. At 15 Days 

**Figure 10:**
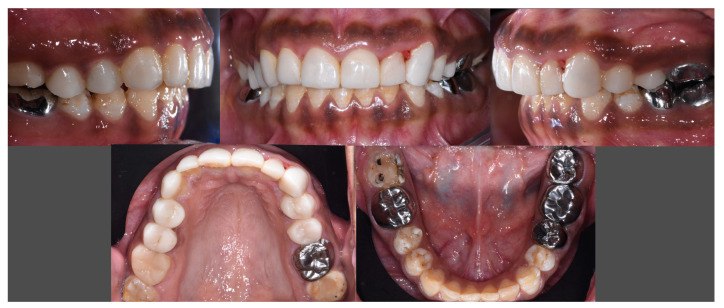
Final intraoral views.

The patient was very satisfied with the aesthetics and reported no pain or sensitivities (Figure 10).

A GOHAI score of 51 was recorded (Table 2).

#### 2.11.2. At 3 Months

Due to the 2020 sanitary crisis, the patient could not be seen.

#### 2.11.3. At 8 Months 

**Figure 11:**
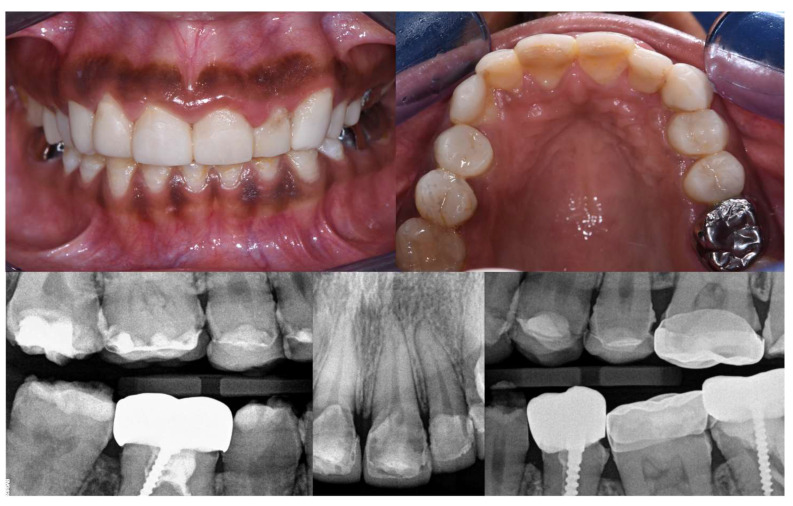
Intraoral views and radiographs at 8-month follow-up.

**Figure 12:**
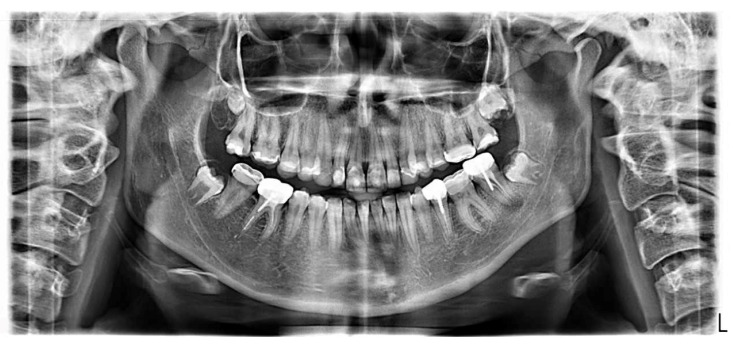
Panoramic radiograph at 8-month follow-up after re-treatment on 47.

The patient was still satisfied with the aesthetic integration of the restorations. She reported a real improvement in wellbeing, with a noticeable decrease in sensitivity, a marked improvement in chewing, and greater self-confidence (Figure 11). The gingiva showed a little bit of healing. No loss of restoration, no marginal discoloration or staining of the material, no chipping, no excessive wear, and no periodontal damage had occurred. Nevertheless, she reported slight sensitivities on the left maxillary canine (23) and the second left maxillary premolar (25) and some pain on the second right mandibular molar (47) (Figure 12). It was decided to apply fluoride on 23 and 25 and to place an SSC on 47. A GOHAI score of 50 was recorded (Table 2).

#### 2.11.4. At One and a Half Years 

**Figure 13:**
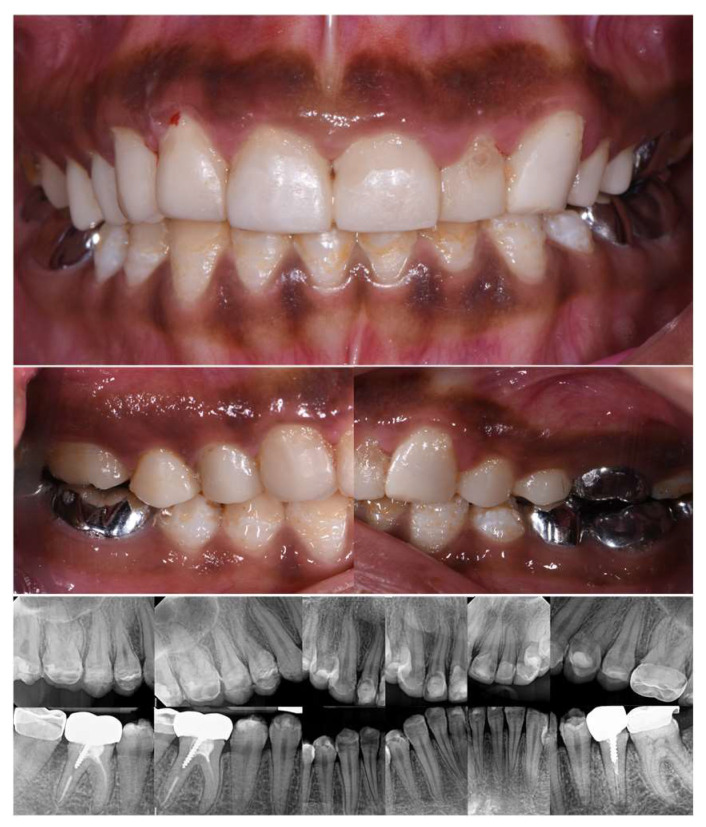
Intraoral views and radiographs at 1.5-year follow-up.

The patient showed bad oral hygiene. This could be explained by the high number of intensive care admissions and their psychological context (Figure 13). She still reported a slight sensitivity on the left maxillary canine (23) and some gingival bleeding during brushing. Nevertheless, she developed oral pain (in the mandibular posterior sectors) and other pains (especially gynecologic). She consulted several departments to find the cause of her body pains. Teeth 46 and 35 had perfectible endodontic treatments that needed to be re-performed. Given the absence of symptoms, monitoring was continued. A GOHAI score of 37 was recorded (Table 2).

#### 2.11.5. At Three Years

**Figure 14:**
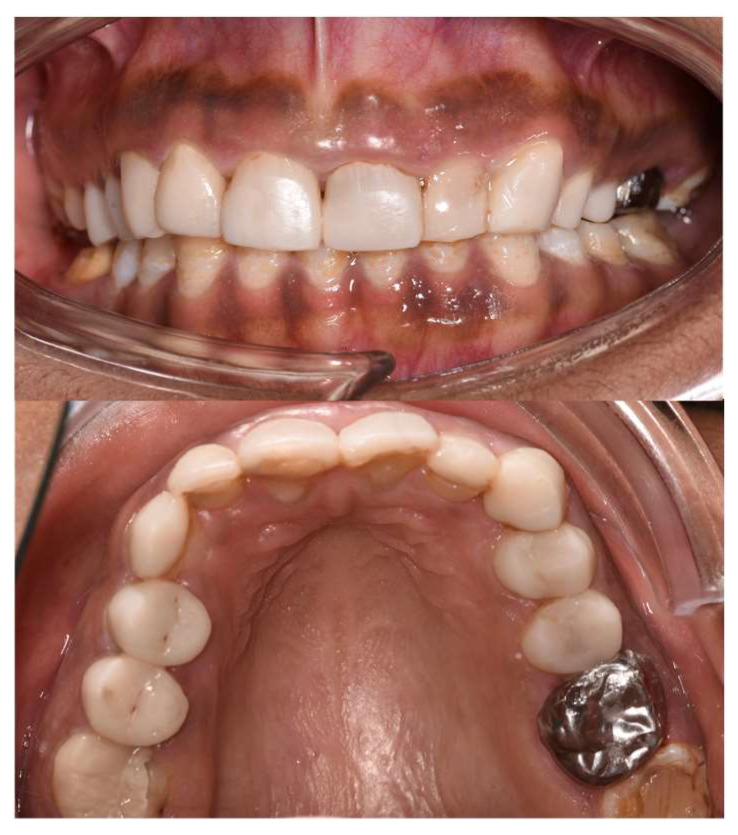
Intraoral views at 3-year follow-up.

The removal of 37, 38, 47, 48, and 18 was decided, along with the endodontic re-treatment of 46 and 35 and new restorations on 35, 36, and 46 (Figure 14). The mandibular posterior teeth needed to be restored in ceramic (lithium disilicate). However, due to several hospitalizations, missing appointments, and compliance difficulties, monitoring was still preferable. Replacement of the maxillary restorations in ceramic was to be scheduled at a later phase. A GOHAI score of 36 was recorded (Table 2).

## 3. Discussion

### 3.1. Hypoparathyroidism and Dental Repercussions

Possible mechanisms causing familial hypoparathyroidism can be associated with the CaSR gene (causing autosomal dominant hypocalcemia), the GCM2 gene (unencoding a transcription factor important for the development, proliferation, and maintenance of the parathyroid cells), the PTH gene (causing impaired secretion of PTH or bioinactive PTH), and variants in the autoimmune regulator (AIRE) gene [11]. Here, the father and the mother of the patient were first cousins, and both had the GCM2 mutation. She had one brother and one half-brother with the GMC2 mutation. She was the only known family member with generalized enamel hypoplasia on permanent teeth, clinically resembling amelogenesis imperfecta. However, according to the mother, the temporary teeth were not affected. To the best of our knowledge, there are no reports in the literature of the general dental management of this type of pathology [15,16,17,18,19,20,21,22,23,24,25].

### 3.2. Digital Wax-Up and Use of CAD-CAM

The implantation of the mock-up key by virtual wax-up, associated with the 3D printing of the model, seemed to be more advantageous than a traditional method by a dental lab technician. The wax-up is essential for programming the treatment plan for the clinician, the prosthetist, and even the family. Indeed, the involvement of the patient and her family at the wax-up step facilitated the requested modifications and made the teenager active in her care process [26]. Thus, at the time of the mock-up fitting, the patient had already validated the future shape and position of her teeth. The clinical mock-up trial was the only way to validate the final aesthetic [27]. Additionally, the use of CAD-CAM avoided the traditional use of unaesthetic metal pediatric preformed crowns for the restorations of molars. Moreover, the digital impression is much more accepted than conventional impression materials and allows for more rapid treatment.

### 3.3. Type of Restorations and Material Selection for the Anterior Teeth

The palatal surface of the maxillary incisors should have been an argument in favor of a total covering using an indirect technique. Nevertheless, in the case of medium-term restorations, to preserve tissue, it was decided to make a palatal return at the most abraded level and to cover the residual palatal parts by adding resin composite using a direct technique. The challenge is to define a threshold age to undertake prosthetic rehabilitations without risking a modification of the gingival position in relation to the defined prosthetic limit [28]. The maturation of the biological space is reached between 16 years (or ten years after the eruption of the molars) and 21 years [29,30,31]. It is therefore advisable to wait until the patient is 20 years of age before the placement of definitive anterior aesthetic restorations to ensure long-term optimal aesthetic integration. Moreover, the patient had a high caries risk and multiple secondary lesions under previous restorations. Reintervention is also easier with a resin composite than with a ceramic material. These arguments supported the choice of direct and indirect resin composite restorations as a medium-term rehabilitation. As we chose a polymer-infiltrated ceramic network for the molars’ restorations for mechanical reasons; we decided to use the same material for the restorations of the anterior teeth, to have homogenous wear and biomechanical deformation [32,33,34,35,36,37,38].

### 3.4. Bonding

No studies exist on resin composite bonding procedures and enamel dysplasia due to hypocalcemia. Nevertheless, this may find some resonance in cases of patients with amelogenesis imperfecta [32,33]. Different strategies have been proposed to overcome the bonding difficulties in this kind of enamel, including the use of 5% sodium hypochlorite for one minute to deproteinize the surface [34]. Adapted enamel–dentin preparation and hard margins are necessary [35]. Except on the margins, bonding was carried out on sound dentin. To optimize the bonding on the enamel margin, we used 5% sodium hypochlorite solution for one minute after etching and before adhesive application. Regarding the dentin, a conventional adhesion protocol was performed because hypochlorite does not significantly increase the bond strength [39,40]. In addition, a universal adhesive seemed particularly indicated because of the patient’s sensitivities [41]. Selective enamel etching was performed in accordance with current recommendations [42]. Overlays and veneers were sequentially bonded with a rubber dam for moisture control: 13 to 23 the first time, followed by 14–15 and 24–25.

### 3.5. Caries Risk Management and Treatment Prognosis

The medium-term follow-up of the patient was difficult and not regular due to her personal context and to the 2021 crisis. We were nevertheless able to conduct a follow-up. The weakest link in this treatment probably resides in the adhesion to the hard tissues and the risk of secondary caries due to management difficulties. After the treatment, tooth sensitivity was reduced and mastication improved, which allowed the patient to achieve better oral hygiene. In addition, the patient was advised about nutrition in limiting the amount and frequency of sugar intake and high-acid foods and drinks, especially between meals [43]. Orthodontic treatment was not considered at this stage because of the patient’s general health problems [44]. It can be proposed later when definitive restorations will be considered.

### 3.6. Quality of Life

Our treatment had a significant psychosocial impact, and the patient reported rapid improvement in quality of life and social and mental wellbeing. In fact, the GOHAI score increased from 28 (low satisfaction) to 51 (average satisfaction) between the start of the treatment and fifteen days after the end of the treatment. After 8 months, the GOHAI score was still quite high, with a score of 50 (average satisfaction). After one and a half years, the GOHAI score decreased to 37 (low satisfaction). After three years, the GOHAI score was 36. Even if the second GOHAI score (first follow-up) did not match a good satisfaction score, the treatment led to an improvement in the patient’s satisfaction, characterized by a true increase in self-confidence and social interaction as shown in the following items with the highest amelioration in dental aspect satisfaction: concern about the oral situation, nervous about the oral situation, and discomfort in eating in public (Table 2).

### 3.7. Limits

The patient did not receive effective follow-up; this was caused by a complicated international general context (the COVID-19 pandemic). This case report concerns only one patient and does not allow for conclusions on the effectiveness of bonding on enamel hypoplasia caused by hypocalcemia.

## 4. Conclusions

Familial hypoparathyroidism with episodes of intrauterine and neonatal hypocalcemia is similar in its management to amelogenesis imperfecta. Thus, as in the latter, the use of direct and indirect resin composites, glass ionomer cements, or posterior SSCs as a conservative treatment option was considered as a viable alternative in medium-term treatment to restore function and aesthetics. Regular follow-up until definitive restoration and regular assessments of quality of life linked to oral health are crucial to ensure the success of the rehabilitation. Other case reports or even case–control studies are necessary to form conclusions on the effectiveness of bonded restorations in this type of patient.

## Figures and Tables

**Table 1:** Classification of causes of hypoparathyroidism.

Congenital Hypoparathyroidism		Acquired Parathyroid Dysfunction	Transient or Functional Hypoparathyroidism
Disorders of Parathyroid Development (Parathyroid Gland Embryogenesis)	Disorders of Parathyroid Function		
Di George syndrome	Activating mutations in the calcium sensing receptor (CaSR)	Post-surgery	Neonatal hypoparathyroidism due to maternal hypercalcemia
APECED syndrome (Autoimmune Poly Endocrinopathy Candidiasis Ectodermal Dystrophy)	Mutation in the PTH gene	Post-radiotherapy	Dysmagnesemia
GCM2 mutation	Mitochondrial diseases (Kearns–Sayre syndrome)	Parathyroid infiltration: hemochromatosis or Wilson disease	Acute alcohol intoxication
HDR syndrome (Hypoparathyroidism Deafness Renal dysplasia)			
HRD syndromes (hypoparathyroidism KCS1 or TBCE or FAM111A mutations)			
Retardation Dysmorphism: X-linked due to SOX3 mutation			

**Table 2:** Patient’s GOHAI answers.

Items	May, 2019	May, 2020	September, 2021	July, 2023
	Before Treatment	After Treatment	At One and a Half Years	At Three Years
Food limitation	Often (2)	Seldom (4)	Seldom (4)	Seldom (4)
Chewing disorder	Often (2)	Never (5)	Sometimes (3)	Sometimes (3)
Comfortable swallowing	Always (5)	Always (5)	Always (5)	Always (5)
Elocution discomfort	Never (5)	Seldom (4)	Sometimes (3)	Sometimes (3)
Alimentation without discomfort	Sometimes (3)	Always (5)	Seldom (2)	Seldom (2)
Social limitation	Often (2)	Jamais (5)	Sometimes (3)	Sometimes (3)
Dental aspect satisfaction	Never (1)	Always (5)	Sometimes (3)	Sometimes (3)
Use of relieve pain medication	Often (2)	Often (2)	Sometimes (3)	Often (2)
Worriness about oral situation	Always (1)	Seldom (4)	Rarement (4)	Rarement (4)
Nervous about oral situation	Always (1)	Never (5)	Often (2)	Often (2)
Discomfort in eating in public	Often (2)	Never (5)	Seldom (4)	Seldom (4)
Teeth sensitivity	Always (1)	Often (2)	Always (1)	Always (1)
**Total score**	**28/60**	**51/60**	**37/60**	**36/60**

## Data Availability

The data presented in this study are available on request from the corresponding author due to ethical reasons.

## References

[1] Pasieka J.L., Wentworth K., Yeo C.T., Cremers S., Dempster D., Fukumoto S., Goswami R., Houillier P., Levine M.A., Pasternak J.D. (2022). Etiology and pathophysiology of hypoparathyroidism: A narrative review. J. Bone Miner. Res..

[2] Hamny I., Chanson P., Borson-Chazot F. (2023). New directions in the treatment of hypoparathyroidism. Ann. Endocrinol..

[3] Liu H., Liu L., Rosen C.J. (2024). PTH and the Regulation of Mesenchymal Cells within the Bone Marrow Niche. Cells.

[4] Fanget F., Demarchi M.S., Maillard L., El Boukili I., Gerard M., Decaussin M., Borson-Chazot F., Lifante J.C. (2021). Hypoparathyroidism: Consequences, economic impact, and perspectives. A case series and systematic review. Ann. Endocrinol..

[5] Reid I.R., Bristow S.M., Bolland M.J. (2015). Calcium supplements: Benefits and risks. J. Intern. Med..

[6] Cooper M., Gittoes N. (2008). Diagnosis and management of hypocalcaemia. BMJ.

[7] Dawale K., Agrawal A. (2022). Parathyroid hormone secretion and related syndromes. Cureus.

[8] Giusti F., Brandi M.L. (2019). Clinical presentation of hypoparathyroidism. Front. Horm. Res..

[9] Zavatta G., Clarke B.L. (2020). Challenges in the management of chronic hypoparathyroidism. Endocr. Connect..

[10] Hejlesen J., Underbjerg L., Gjørup H., Bloch-Zupan A., Sikjaer T., Rejnmark L., Haubek D. (2019). Dental findings in patients with non-surgical hypoparathyroidism and pseudohypoparathyroidism: A systematic review. Front. Physiol..

[11] Loe H., Theilade E., Jensen S.B. (1965). Experimental gingivitis in man. J. Periodontol..

[12] Angle E.H. (1903). Some Basic Principles in Orthodontia. Int. Dent. J..

[13] Velázquez-Olmedo L., Ortíz-Barrios L., Cervantes-Velazquez A., Cárdenas-Bahena A., García-Peña C., Sánchez-García S. (2014). Quality of life related to oral health in older people. Evaluation instruments. Rev. Med. Inst. Mex. Seguro Soc..

[14] Arai T., Kinoshita S. (1977). A comparison of plaque removal by different toothbrushes and toothbrushing methods. Bull. Tokyo Med. Dent. Univ..

[15] Hendy G.N., Cole D.E., Bastepe M., Feingold K.R., Anawalt B., Blackman M.R., Boyce A., Chrousos G., Corpas E., de Herder W.W., Dhatariya K., Dungan K., Hofland J. (2017). Hypoparathyroidism and Pseudohypoparathyroidism. Endotext.

[16] Arnaud C., Maijer R., Reade T., Scriver C.R., Whelan D.T. (1970). Vitamin D dependency: An inherited postnatal syndrome with secondary hyperparathyroidism. Pediatrics.

[17] Stimmler L., Snodgrass G.J.A.I., Jaffe E. (1973). Dental defects associated with neonatal symptomatic hypocalcaemia. Arch. Dis. Child..

[18] Levine R.S., Keen J.H. (1974). Neonatal enamel hypoplasia in association with symptomatic neonatal hypocalcaemia. Br. Dent. J..

[19] Norén J. (1984). Microscopic study of enamel defects in deciduous teeth of infants of diabetic mothers. Acta Odontol. Scand..

[20] Ranggård L., Östlund J., Nelson N., Norén J. (1995). Clinical and histologic appearance in enamel of primary teeth from children with neonatal hypocalcemia induced by blood exchange transfusion. Acta Odontol. Scand..

[21] Ranggård L. (1994). Dental enamel in relation to ionized calcium and parathyroid hormone. Studies of human primary teeth and rat maxillary incisors. Swed. Dent. J. Suppl..

[22] Klingberg G., Dietz W., Óskarsdóttir S., Odelius H., Gelander L., Norén J. (2005). Morphological appearance and chemical composition of enamel in primary teeth from patients with 22q11 deletion syndrome. Eur. J. Oral Sci..

[23] Kelly A., Pomarico L., de Souza I. (2009). Cessation of dental development in a child with idiopathic hypoparathyroidism: A 5-year follow-up. Oral Surg. Oral Med. Oral Pathol. Oral Radiol. Endod..

[24] Moussaid Y., Griffiths D., Richard B., Dieux A., Lemerrer M., Léger J., Lacombe D., Bailleul-Forestier I. (2012). Oral manifestations of patients with Kenny–Caffey Syndrome. Eur. J. Med. Genet..

[25] Hejlesen J., Underbjerg L., Gjørup H., Sikjaer T., Rejnmark L., Haubek D. (2019). Dental anomalies and orthodontic characteristics in patients with pseudohypoparathyroidism. BMC Oral Health.

[26] Lv L., He W., Ye H., Cheung K., Tang L., Wang S., You L., Xun C., Zhou Y. (2022). Interdisciplinary 3D digital treatment simulation before complex esthetic rehabilitation of orthodontic, orthognathic and prosthetic treatment: Workflow establishment and primary evaluation. BMC Oral Health.

[27] Revilla-León M., Besné-Torre A., Sánchez-Rubio J., Fábrega J., Özcan M. (2018). Digital tools and 3D printing technologies integrated into the workflow of restorative treatment: A clinical report. J. Prosthet. Dent..

[28] Gargiulo A.W., Wentz F.M., Orban B. (1961). Dimensions and relations of the dentogingival junction in humans. J. Periodontol..

[29] Gottlieb B. (1927). The gingival margin. Proc. R. Soc. Med..

[30] Moshrefi A. (2000). Altered passive eruption. J. West Soc. Periodontol. Periodontal Abstr..

[31] Weinberg M.A., Eskow R.N. (2000). An overview of delayed passive eruption. Compend. Contin. Educ. Dent..

[32] Yu H., Özcan M., Yoshida K., Cheng H., Sawase T. (2020). Bonding to industrial indirect composite blocks: A systematic review and meta-analysis. Dent. Mater..

[33] Abdou A., Takagaki T., Alghamdi A., Tichy A., Nikaido T., Tagami J. (2021). Bonding performance of dispersed filler resin composite CAD/CAM blocks with different surface treatment protocols. Dent. Mater. J..

[34] Eldafrawy M., Greimers L., Bekaert S., Gailly P., Lenaerts C., Nguyen J.F., Sadoun M., Mainjot A. (2019). Silane influence on bonding to CAD-CAM composites: An interfacial fracture toughness study. Dent. Mater..

[35] Eldafrawy M., Ebroin M.G., Gailly P.A., Nguyen J.F., Sadoun M.J., Mainjot A. (2018). Bonding to CAD-CAM composites: An interfacial fracture toughness approach. J. Dent. Res..

[36] Strauch S., Hahnel S. (2018). Restorative treatment in patients with amelogenesis imperfecta: A review. J. Prosthodont..

[37] Dursun E., Savard E., Vargas C., Loison-Robert L., Cherifi H., Bdeoui F., Landru M.M. (2016). Management of amelogenesis imperfecta: A 15-year case history of two siblings. Oper. Dent..

[38] Sönmez I.S., Aras S., Tunç E.S., Küçükeşmen C. (2009). Clinical success of deproteinization in hypocalcified amelogenesis imperfecta. Quintessence Int..

[39] Krämer N., Bui Khac N., Lücker S., Stachniss V., Frankenberger R. (2018). Bonding strategies for MIH-affected enamel and dentin. Dent. Mater..

[40] Hardan L., Bourgi R., Kharouf N., Mancino D., Zarow M., Jakubowicz N., Haikel Y., Cuevas-Suárez C.E. (2021). Bond strength of universal adhesives to dentin: A systematic review and meta-analysis. Polymers.

[41] Hong X., Huang Z., Tong Z., Jiang H., Su M. (2021). Clinical effects of different etching modes for universal adhesives: A systematic review and meta-analysis. Ann. Palliat. Med..

[42] Szesz A., Parreiras S., Reis A., Loguercio A. (2016). Selective enamel etching in cervical lesions for self-etch adhesives: A systematic review and meta-analysis. J. Dent..

[43] Moores C.J., Kelly S.A.M., Moynihan P.J. (2022). Systematic review of the effect on caries of sugars intake: Ten-year update. J. Dent. Res..

[44] Ata-Ali F., Ata-Ali J., Ferrer-Molina M., Cobo T., De Carlos F., Cobo J. (2016). Adverse effects of lingual and buccal orthodontic techniques: A systematic review and meta-analysis. Am. J. Orthod. Dentofacial Orthop..

